# N7-Methylguanosine Regulatory Genes Profoundly Affect the Prognosis, Progression, and Antitumor Immune Response of Hepatocellular Carcinoma

**DOI:** 10.3389/fsurg.2022.893977

**Published:** 2022-06-16

**Authors:** Kexiang Zhou, Jiaqun Yang, Xiaoyan Li, Wei Xiong, Pengbin Zhang, Xuqing Zhang

**Affiliations:** ^1^ Department of Gastroenterology, The Third Affiliated Hospital of ChongQing Medical University, China; ^2^ ChongQing Medical University, Chongqing, China; ^3^ Department of Infectious Diseases, The Third Affiliated Hospital of ChongQing Medical University, China

**Keywords:** hepatocellular carcinoma, N7-methylguanosine, risk signature, prognosis, tumor immune microenvironment, NCBP2

## Abstract

**Background:**

Hepatocellular carcinoma (HCC) is a common abdominal cancer with poor survival outcomes. Although there is growing evidence that N7-methylguanosine (m7G) is closely associated with tumor prognosis, development, and immune response, few studies focus on this topic.

**Methods:**

The novel m7G risk signature was constructed through the Lasso regression analysis. Its prognostic value was evaluated through a series of survival analyses and was tested in ICGC-LIRI, GSE14520, and GSE116174 cohorts. CIBERSORT, ssGSEA, and ESTIMATE methods were applied to explore the effects of the m7G risk score on tumor immune microenvironment (TIM). The GSEA method was used to evaluate the impacts of the m7G risk score on glycolysis, ferroptosis, and pyroptosis. The human protein atlas (HPA) database was used to clarify the histological expression levels of five m7G signature genes. The biofunctions of NCBP2 in hepatocellular cancer (HC) cells were confirmed through qPCR, CCK8, and transwell assays.

**Results:**

Five m7G regulatory genes comprised the novel risk signature. The m7G risk score was identified as an independent prognostic factor of HCC and could increase the decision-making benefit of traditional prognostic models. Besides, we established a nomogram containing the clinical stage and m7G risk score to predict the survival rates of HCC patients. The prognostic value of the m7G model was successfully validated in ICGC and GSE116174 cohorts. Moreover, high m7G risk led to a decreased infiltration level of CD8+ T cells, whereas it increased the infiltration levels of Tregs and macrophages. The glycolysis and pyroptosis processes were found to be enriched in the HCC patients with high m7G risk. Finally, overexpression of NCBP2 could promote the proliferation, migration, and invasion of HC cells.

**Conclusions:**

The m7G risk score was closely related to the prognosis, antitumor immune process, glycolysis, and malignant progression of HCC. NCBP2 has pro-oncogenic abilities, showing promise as a novel treatment target.

## Introduction

Hepatocellular carcinoma (HCC) is a common digestive tumor, accounting for 6% of all cancer-related deaths in the United States (1). Despite considerable advances in its diagnosis and treatment, the overall survival (OS) is still unsatisfactory. Especially in China, the region having the largest number of HCC patients in the world, the median survival of patients is commonly less than 30 months (2). Regretfully, the effects of existing therapeutic approaches are unsatisfactory. Sorafenib is the first-line option for treating advanced HCC; the median OS of patients who receive sorafenib is only 18 months (3). Nivolumab, another commonly used immune checkpoint blockade (ICB) for HCC systemic treatment, only confers HCC patients with a median OS of 15 months (4). Meanwhile, its objective response rate (ORR) is less than 20% (5). Therefore, finding novel therapeutic strategies and establishing an accurate prognostic system are imperative.

RNA epigenetic modifications have been confirmed to be closely involved in the onset and progression of multiple cancers (6). One classic example is the N6-methyladenosine (m6A) modification. Ample evidence indicates that the m6A process profoundly affects the prognosis, tumor immune microenvironment (TIM), and therapeutic effects of various cancers, including adrenocortical carcinoma (ACC) (7), pancreatic adenocarcinoma (PAAD) (8, 9), lung adenocarcinoma (LADC) (10), HCC (11), *etc*. Unfortunately, extremely limiting research focuses on the N7-methylguanosine (m7G), which is another one of the most prevalent RNA modifications. m7G refers to the modification of the seventh N of RNA guanine with a methyl group (12). Mechanistically, this guanosine methylation at the 5′ cap of RNA relies on the catalyzation of METTL1 and its co-factor WD repeat domain 4 (WDR4) (13). Since m7G modification can improve the stability of mRNA, this process is speculated to mediate several biological functions through altering the expression levels of related genes (14, 15). Recently, Dai et al. found that m7G RNA modification could enhance oncogenic mRNA translation and promote intrahepatic cholangiocarcinoma (ICC) progression (16). Therefore, m7G, an unfamiliar epigenetic topic, has great potential to advance the paradigm of HCC treatment and clinical assessment.

To the best of our knowledge, no research has investigated the associations of m7G regulatory genes with the prognosis and immune response of cancers. Therefore, we constructed a novel m7G risk signature for the first time through the Lasso regression analysis. The roles of m7G risk score in prognosis, progression, TIM, and glycolysis metabolism of HCC were explored. Moreover, the biofunctions of NCBP2, a critical member of the m7G risk signature, were also confirmed through the experiments in vitro. It is conceivable that our findings could bring new insights into the treatment and prognosis assessment of HCC.

## Materials and Methods

### Data Source

The clinical information and gene expression data were derived from TCGA (https://portal.gdc.cancer.gov/), ICGC (https://dcc.icgc.org/releases), and GEO (https://www.ncbi.nlm.nih.gov/geo/) databases. Given that the number of normal liver samples in TCGA (*n* = 50) did not well match that of HCC samples, we used 110 normal liver tissue samples from the GTEx database (https://xenabrowser.net/datapages/) to solve this shortage. The enrolled TCGA samples should provide both the gene expression matrix and the corresponding clinical information. Besides, 28 TCGA samples were excluded owing to their too short follow-up (less than 30 days). To minimize the batch effect between different datasets, we used the “ComBat” algorithm to correct it (17), which was implemented by the SVA package in R software. The normalized batch-corrected plots of TCGA-LIHC, ICGC-LIRI, GSE14520, and GSE116174 cohorts are shown in Supplementary Figure S1. Transcriptome data were standardized by log2 (FPKM + 1) transformation. The clinical characteristics of TCGA, ICGC, and GEO cohorts are presented in Supplementary Tables S1, S2.

### m7G-related Gene Set

In the present study, we established an m7G gene set using the Molecular Signatures Database (MSigDB) (https://www.gsea-msigdb.org/gsea/msigdb/). As mentioned in some m7G-related reviews, this RNA modification refers to the methylation of the seventh guanosine at the 5′ end of RNA. In this context, we screened out three core m7G-related gene sets from the MSigDB database, including “GOMF RNA 7-Methylguansosine Cap Binding,” “GOMF m7G 5-PPPN Diphosphatase Activity,” and “GOMF RNA Cap Binding.” The detailed description of these gene sets is shown in Supplementary Table S3. Using these three MSigDB gene sets, we constructed a comprehensive m7G gene set, whose gene members are presented in Supplementary Table S4.

### Construction of m7G Risk Signature

The m7G differentially expressed genes (DEGs) were screened out by the “Limma” package in R software (Ver. 3.6.3). The threshold for the screening criterion was set at adjusted *P*-value <0.05 and the absolute value of log_2_FC ≥ 0.58 (1.5-fold difference in gene expression). The m7G regulatory genes with prognostic values were identified by cox univariate regression analysis. The intersection part between DEGs and prognostic genes was selected by the Venn diagram. Finally, these intersection genes were applied to construct a novel m7G risk signature through the Lasso regression analysis.

### Evaluation of the Prognostic Value

The m7G risk score of each HCC patient was calculated in terms of the novel risk signature. The Cutoff Finder online tool (http://molpath.charite.de/cutoff) was used to determine the optimal cutoff value of the m7G risk score (18). According to the value, patients were divided into high- and low-risk groups. Survival differences between different risk groups were compared based on the Kaplan–Meier method. Cox univariate and multivariate analyses were applied to identify the independent prognostic factors. The predictive accuracy of the m7G risk signature was evaluated by the receiver operating characteristic curve (ROC). Decision curve analysis (DCA) was used to determine whether the m7G risk score could improve the clinical decision-making benefit of two traditional prognostic models of HCC. The traditional model A was composed of age, histological grade, and TNM staging. The traditional model B was composed of age, histological grade, and clinical stage. Except for the M1 stage (*n* = 4), the clinical subgroup analyses were performed to assess the application scope of the m7G risk signature. A nomogram was constructed to predict the OSR of an individual at 1, 3, and 5 years. Besides, GSE14520 (*n* = 221), GSE116174 (*n* = 64) and ICGC-LIRI (*n* = 221) datasets as the validation cohorts were employed to further test the prognostic value of the m7G model.

### Immune Analysis

The immune abundance of 21 lymphocyte subtypes in each HCC sample was calculated using the CIBERSORT algorithm (19). The activities of 10 immune-related pathways were calculated based on the ssGSEA (single-sample gene set enrichment analysis) method (20). The ESTIMATE algorithm is an approach to quantify the immune cell admixture in tumor parenchyma and stroma, by which the tumor immune microenvironment (TIM) could be depicted to some extent (21). The scores of stromal, immune, and ESTIMATE in different risk groups were calculated.

### GSEA

GSEA (Gene Set Enrichment Analysis) was utilized to analyze the impacts of m7G risk levels on the glycolysis process, ferroptosis, and pyroptosis. The used gene sets were obtained from the MsigDB database, including “GO Glycolytic Process,” “Hallmark Glycolysis,” “Reactome Pyroptosis,” and “WP Ferroptosis.” Phenotype labels were set as high-m7G risk samples versus low-m7G risk ones. The number of permutations was set as 1,000. There was no collapse in gene symbol. The descriptions of these gene sets are given in Supplementary Table S5.

### HPA Database

The human protein atlas (HPA) database exhibits the landscape of cancer proteomes in 32 different tissues and organs (https://www.proteinatlas.org/) (22). Using the HPA database, we confirmed the histological expression levels of m7G signature genes between normal liver and HCC tissues.

### Cell Culture and Transfection

Two human hepatocellular cancer cell lines (HepG2 and Huh-7) were purchased from Procell Life Science&Technology Company (Wuhan, China). One normal liver cell line (THLE-3) was purchased from Otwo Biotechnology (Guangzhou, China). HepG2 and THLE-3 cells were both cultured in MEM (minimum essential medium) containing 10% FBS (fetal bovine serum) and 1% P/S (penicillin/streptomycin) (Procell, Wuhan, China). Huh-7 cells were cultured in DMEM (Dulbecco’s modified Eagle’s medium) containing 10% FBS and 1% P/S (Procell, Wuhan, China). Specific siRNA and amplification plasmids were designed by HanHeng Biotechnology (Shanghai, China). Lentiviruses (HanHeng Biotechnology, Shanghai, China) are used to transfect liver cancer (LC) cells.

### Clinical Samples and RT-qPCR

We collected 15 pairs of HCC and adjacent normal tissues from the Third Affiliated Hospital of Chongqing Medical University. qPCR and western blot tests were employed to confirm the differential expression of NCBP2 in HCC specimens. We received informed consent from all patients. The study protocol was approved by the Ethics Committees of the Third Affiliated Hospital of Chongqing Medical University.

Total RNA was extracted using TRIzol reagent (Thermo Fisher Scientific, Waltham, MA, USA). Reverse transcription (RT) was performed using the PrimeScript RT reagent kit with gDNA eraser (Takara, Japan). Transcript levels were measured using the SYBR-Green PCR reagent (Takara, Japan). Then, RT-qPCR was performed on an ABI Prism 7900 sequence detection system. GAPDH was used as an internal reference. mRNA expression was calculated based on the 2^−ΔΔCT^ method. The primer list is presented in Supplementary Table S6.

### Western Blot

The detailed manipulations were similar to those previously described (23). After washing with PBS twice, transfected cells were lysed on ice by RIPA buffer (Beyotime, China). Centrifugation was performed at 12,000 rpm for 4 min. Then, the supernatant was collected and moved into an EP tube. The protein concentration was measured by the BCA kit (Phygene Life Sciences Company, Fuzhou, China). Sample absorbance at 562 nm was measured after 30 min incubation with the BCA working reagent. The protein concentration of each sample was calculated according to the standard curve. Sample proteins were separated by 10% SDS-PAGE. After electrophoresis, protein samples were transferred to PVDF membranes (BestBio, Shanghai, China). The PVDF membranes were blocked by 5% skim milk at 37°C for 2 h and then were washed with TBST buffer (BIOSIC, Nanjing, China) three times. The treated membranes were incubated with the primary antibody overnight at 4°C. After washing again with TBST three times, the membranes were incubated with the secondary antibody. The primary and secondary antibodies were both purchased from Abcam Scientific (Shanghai, China). The primary antibodies were as follows: rabbit polyclonal anti-NCBP1 (anti-CBP80) (1:1000, ab154532) and rabbit polyclonal anti-GAPDH antibody (1:1,000, ab22555). The secondary antibody was the goat-anti rabbit IgG-HRP secondary antibody (1:2000, ab205718).

### CCK8 Assay

Cell counting kit-8 assays (CCK-8, Dojindo, Tokyo, Japan) were employed to evaluate the viability of HepG2 and HuH-7 cells. Transfected cells were plated into 96-well plates with a density of 5 × 10^3^ cells per well and incubated overnight at 37°C. At each observed time point, the cells were incubated with 10 μL of CCK-8 reagent for 2 h at 37°C. The absorbance of each well was detected at 450 nm by a spectrophotometer.

### Transwell Migration and Invasion Assays

Transwell migration and invasion assays were carried out to assess the malignant behaviors of LC cells. The experiment procedure was as described in a previous study (7). Transfected cells (2 × 10^4^ per well) were placed into 24-well Transwell chambers (Corning, NY, USA). DMEM or MEM medium containing 0.1% FBS was added to upper chambers, while that with 10% FBS was added to the lower ones. After 48 h incubation, the migratory cells were fixed by paraformaldehyde and stained with 0.1% crystal violet. Cell counting was performed under a high-magnification microscope (100-fold) from five random visual fields. For invasion assays, the upper chambers were precoated with Matrigel.

## Results

### Novel m7G Risk Signature Is Constructed Through the Lasso Regression Analysis

**Figure 1:**
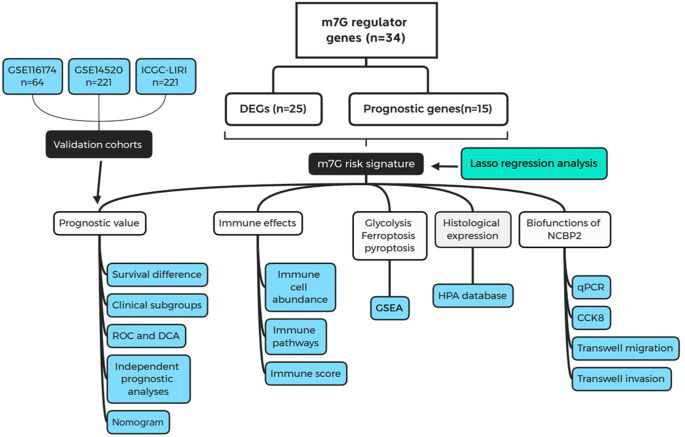
Flow chart of the present study. m7G, N7-methylguanosine; DEGs, differentially expressed genes; ROC, receiver operating characteristic curve; DCA, decision curve analysis; GSEA, gene set enrichment analysis; HPA, Human Protein Atlas.

**Figure 2:**
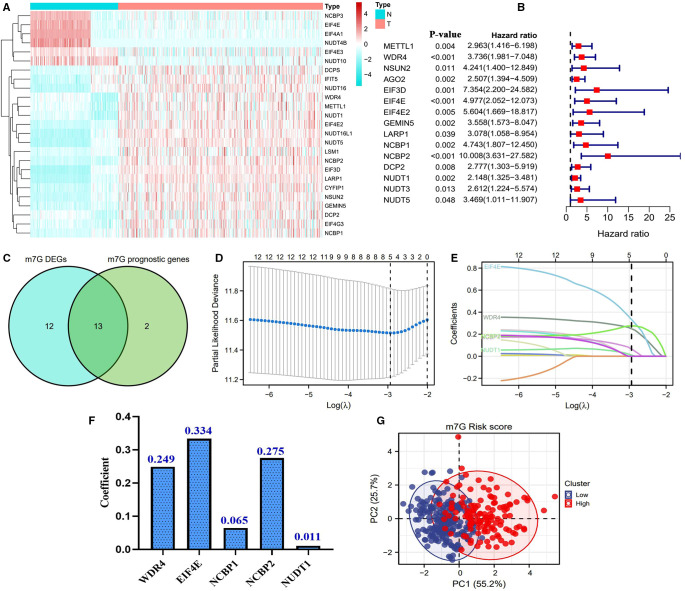
Construction of a novel m7G risk signature. (**A**) Heatmap of m7G DEGs. Blue represents downregulated expression, and red represents upregulated expression. The bar on the left side represents the expressive Z-score. (**B**) Identification of prognostic m7G genes through cox univariate analyses. (**C**) Venn plots showing the intersection of genes between DEGs and prognostic m7G genes. (**D,E**) Process of the Lasso regression analysis. (**F**) Coefficients of each member gene in the m7G risk signature. (**G**) PCA plots of the m7G risk score. Survival status is used as the outcome measure. The m7G risk level is used as the clustering criterion.

The flowchart of this study is presented in Figure 1. Most of the m7G regulatory genes (73.5%, 25/34) are differentially expressed in HCC samples. Among them, NUDT4B, EIF4A1, NUDT10, EIF4E, NCBP3, and EIF4E3 were downregulated, while others were upregulated (Figure 2A). Meanwhile, 15 m7G genes were found to have abilities to affect the prognosis of HCC patients (Figure 2B). Interestingly, all these prognostic genes were risk factors for the poor prognosis. Finally, a total of 13 m7G genes were screened out to construct the risk signature (Figure 2C). Through the Lasso regression analysis (Figure 2DE), we established a novel m7G risk signature that comprised five members. The m7G risk score of each HCC patient was calculated according to the following formula (Figure 2F): m7G risk score = 0.249 *  (WDR4 relative expression) + 0.334 *  (EIF4E relative expression) + 0.065 *  (NCBP1 relative expression) + 0.275 *  (NCBP2 relative expression) + 0.011 *  (NUDT1 relative expression). According to the optimal cutoff value of risk score (1.912), 442 HCC patients were divided into high- and low-risk groups. The m7G risk plots are exhibited in Supplementary Figure S2. Moreover, the m7G risk score could explain the 80.9% of the prognostic variation (Figure 1G), suggesting that the m7G risk signature had outstanding abilities to discriminate the HCC patients with different survival outcomes.

### m7G Risk Signature Provides Crucial Prognostic Information

**Figure 3:**
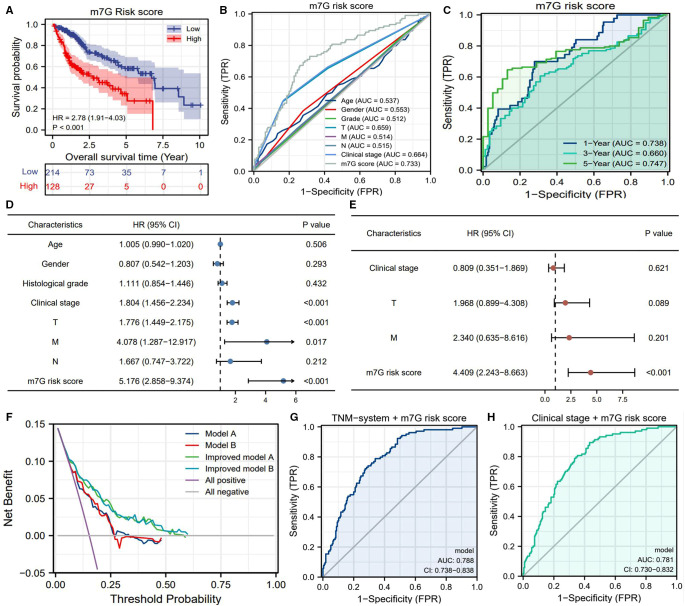
Prognostic value of m7G risk signature in HCC. (**A**) Overall survival difference between the high- and low-m7G-risk groups based on the Kaplan–Meier method. (**B**) ROC curves of clinicopathological features and m7G risk scores for predicting OSR. (**C**) Time-dependent ROC curves of the m7G risk score for predicting OSR. (**D**) Univariate independent prognostic analysis. (**E**) Multivariate independent prognostic analysis. (**F**) DCA results. “Model A” is the traditional prognostic model composed of age, histological grade, and TNM staging (blue line). “Model B” is the traditional prognostic model composed of age, histological grade, and clinical stage (red line). “Model C” is the improved model A with the m7G risk score (green line). “Model D” is the improved model B with the m7G risk score (light blue line). (**G–H**) Improvements brought by the m7G risk score in the predictive accuracy of TNM staging and clinical stage. OSR, overall survival rate; HR, hazards ratio; AUC, area under curve; TPR, true-positive rate; FPR, false-positive rate.

High m7G risk resulted in a poor prognosis (HR = 2.78, *P *<* *0.01) (Figure 3A). In contrast to the clinicopathological features of HCC, the m7G risk score possessed an excellent predicted accuracy (AUC = 0.733) (Figure 3B). Besides, it had the highest accuracy for predicting the five-year survival rate (AUC = 0.747) (Figure 3C). Through the cox regression analyses, only the m7G risk score was identified as the independent prognostic factor of HCC (Figures 3D,E). More importantly, DCA plots revealed that the m7G risk score greatly increased the clinical-decision benefit of two traditional prognostic models (Figure 3F). Meanwhile, the combination of the m7G risk score with either TNM staging or AJCC stage could significantly improve the predicted accuracy of the previous prognostic system (Figures 3G,H vs. 3B).

**Figure 4:**
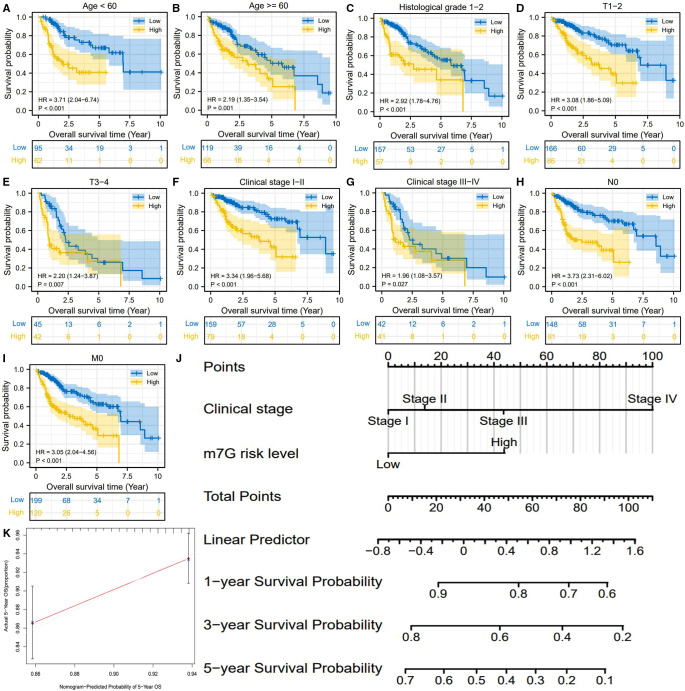
m7G risk signature is available for most of the clinical subgroups. (**A–I**) Overall survival difference between high- and low-m7G-risk groups in each clinical subgroup. (**J**) Nomogram is used for predicting 1-, 3-, 5-year overall survival probability of HCC patients. (**K**) Calibration curves for evaluating the predictive accuracy of the m7G nomogram.

We also observed that the m7G risk score is equipped with a wide range of applicability. The m7G risk signature could distinguish the survival differences of HCC patients in most of the clinical subgroups (Figures 4A–I). For advancing clinical practice, we constructed a nomogram that consisted of clinical stage and m7G risk level (Figure 4J). The calibration plot showed that the predicted probabilities well-matched with actual survival rates (Figure 4K).

### m7G risk Signature Is Also Applicable to Validation Cohorts

**Figure 5:**
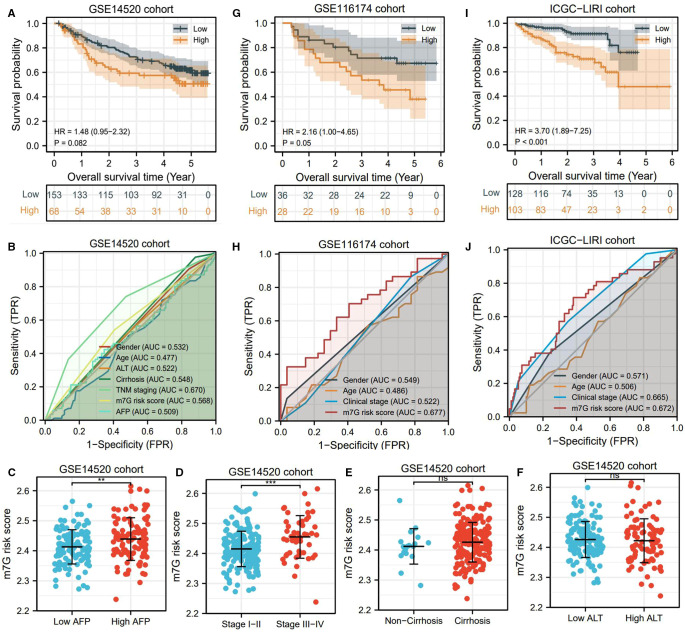
m7G risk signature is successfully tested in validation cohorts. (**A,B**) Survival difference and ROC analyses in the GSE14520 cohort. (**C–F**) Differences in m7G risk scores between different clinical markers in the GSE14520 cohort. (**G,H**) Survival differences and ROC analyses in the GSE116174 cohort. (**I,J**) Survival differences and ROC analyses in the ICGC-LIRI cohort. *P < 0.05, **P < 0.01, ***P < 0.001.

Using three validation cohorts, we tested the prognostic value of the m7G risk signature. In the GSE14520 cohort, although the overall survival rates between different risk groups presented a certain difference, it was not statistically significant (HR = 1.48, *P *= 0.082) (Figure 5A). The predicted accuracy of the m7G risk score in GSE14520 was around 0.5 but was still higher than most HCC clinical indicators (Figure 5B). Besides, the m7G risk score in the patients with high AFP levels and advanced clinical stages was much higher than that in the patients with low AFP levels and local cases (Figure 5C,D). However, the m7G risk score was not associated with the clinical history of cirrhosis and ALT level (Figures 5E,F). Different findings were observed in GSE116174 and IGCG-LIRI cohorts. In these two cohorts, high risk conferred unfavorable survival outcomes (Figures 5G,I). Moreover, the m7G risk score was superior in predicting prognosis compared with the age and clinical stage (Figures 5H,J). Overall, the m7G risk signature was successfully validated in two external cohorts.

### High m7G Risk Is Detrimental to the Anticancer Immune Process

**Figure 6:**
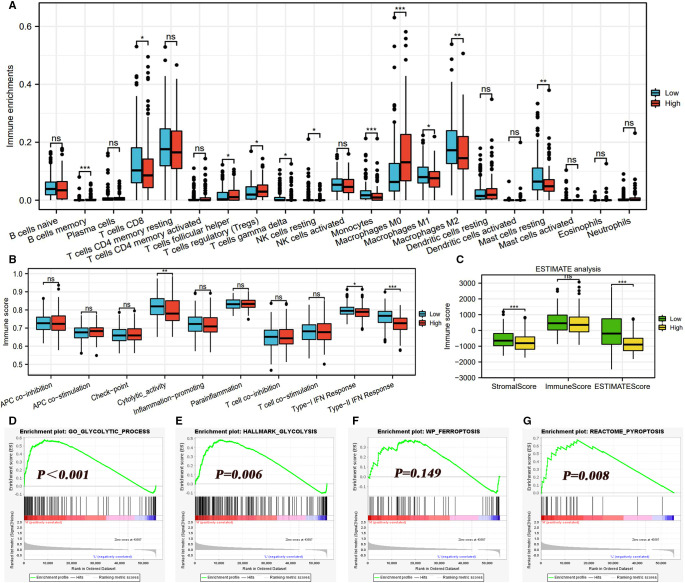
Effects of the m7G risk score on TIM. (**A**) Differences in infiltrating levels of 21 immune cells between high- and low-m7G-risk groups based on the CIBERSORT algorithm. High m7G risk is red, and low m7G risk is blue. (**B**) Differences in activities of 10 immune-related pathways between different m7G risk groups based on the ssGSEA algorithm. (**C**) Differences in immune scores between different m7G risk groups based on the ESTIMATE method. (**D–G**) Effects of m7G risk levels on the enrichments of glycolysis, ferroptosis, and pyroptosis based on GSEA analyses. TIM, tumor immune microenvironment; GSEA, gene set enrichment analysis; *P < 0.05, **P < 0.01, ***P < 0.001.

The infiltrating abundances of 21 immune cells in each HCC sample are presented in Supplementary Figure S3. High m7G risk significantly reduced the infiltrating levels of CD8 T cells, resting NK cells, gamma delta T cells, monocytes, M1macrophages, M2macrophages, and resting mast cells; inversely, it increased that of follicular helper T cells, regulatory T cells (Tregs), and macrophages M0 (Figure 6A). As shown in Table 1, most of the alterations of immune abundances suppressed the antitumor process and promoted the formation of the immunotolerant microenvironment. As for immune-related pathways, high m7G risk was accompanied by the low activities of cytolytic activity and type-II IFN (interferon) response (Figure 6B). ESTIMATE analyses showed that stromal and ESTIMATE scores in the high-risk group were all significantly lower than those in the low-risk group (Figure 6C), which indicated that high m7G risk was difficult to activate the antitumor immunity.

**Table 1:** Effects of m7G risk levels on tumor immune microenvironment.

Immune cell	Variation trend in high m7G risk	Roles in tumor immunity	Final effect on antitumor immunity
CD8T cells	Decreased	CD8+ T cells exert potent cytotoxic effects to eradicate tumour cells	Unfavorable
Follicular helper T cells	Increased	TFH is a subset of CD4+ T cells specialized to prevent excessive antibody response	Unfavorable
Regulatory T cells	Increased	Tregs are capable of suppressing the functions of CD8+ T cells	Unfavorable
Gamma delta T cells	Decreased	The gamma delta T cells have capacities for killing tumor cells	Unfavorable
M0 macrophages	Increased	The polarization of macrophage M1/M2 results in different immune responses	Uncertain
M1 macrophages	Decreased	M1 macrophages can phagocytose tumor cells through their proinflammatory abilities	Unfavorable
M2 macrophages	Decreased	M2 macrophages can promote tumor growth and invasion though their anti-inflammatory abilities	Favorable

*m7G, N7-methylguanosine; TFH, follicular helper T cells; Tregs, regulatory T cells.*

### m7G Risk Score Is Associated With the Glycolysis and Pyroptosis

Glycolysis is the main hallmark of cancer metabolism. In view of this, we investigated the effects of the m7G risk score on the enrichments of the glycolytic process. As expected, glycolysis was significantly enriched in the HCC samples with high m7G risk (Figures 6D,E). Interestingly, m7G risk levels failed to affect the enrichment of ferroptosis (Figure 6F), whereas high m7G risk could stimulate pyroptosis (Figure 6G), which revealed that different patterns of programmed cell death (PCD) acted different roles in m7G-mediated HCC progression.

### m7G Signature Genes Differentially Express in Hepatocellular Carcinoma Tissues

**Figure 7:**
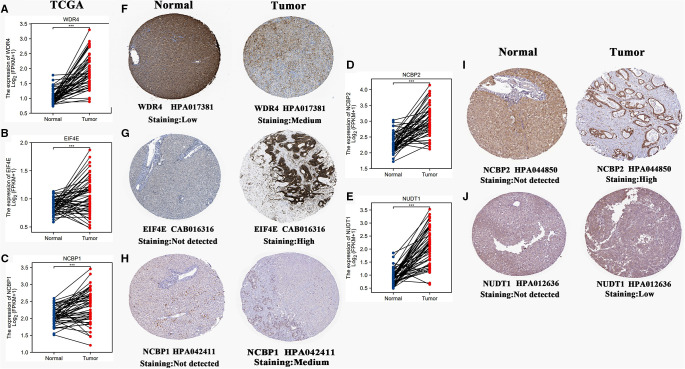
Histological expressions of five m7G signature genes. (**A–E**) Differences in mRNA expressions of 5 m7G genes between normal and HCC samples based on the TCGA-LIHC cohort. (**F–J**) Differences in histological expressions of 5 m7G genes between normal and HCC tissues based on the HPA database. The top of the figure indicates the category of the tissue specimen. The name of the m7G signature gene, the antibody type, and the staining intensity are listed at the bottom of each image. *P < 0.05, **P < 0.01, ***P < 0.001.

The mRNA expression levels of five m7G signature genes were all significantly upregulated in HCC tissues compared to normal liver tissues (Figures 7A–E). Using the HPA database, we investigated the histological expression levels of these genes (Figures 7F–J). All these signature genes presented low or nondetectable expression in normal tissues. In tumor tissues, EIF4E and NCBP2 were highly expressed, while WDR4 and NCBP1 were moderately expressed. Despite low expression of NUDT1 in tumor tissues, NUDT1 was not detectable in normal ones.

### NCBP2 Has Cancer-Promoting Capacities in Hepatoma Cells

**Figure 8:**
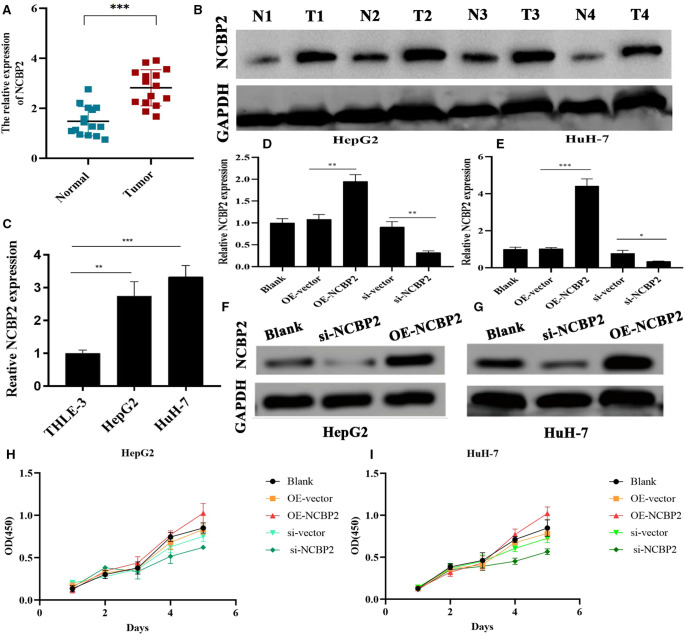
Overexpression of NCBP2 promotes the proliferation of LC cells. (**A**) RT-qPCR detection on 15 pairs of clinical samples confirmed the differential mRNA expressions of NCBP2 between adjacent normal and HCC tissues. (**B**) Western blot results for four pairs of clinical samples confirming the differential protein expressions of NCBP2. (**C**) Expressive differences of NCBP2 between normal liver and LC cells. (**D,E**) Transfection efficiency of si-NCBP2 and OE-NCBP2 in HepG2 and HuH-7 cells based on RT-qPCR tests. (**F,G**) Effects of si-NCBP2 and OE-NCBP2 on protein expression of NCBP2 in LC cells based on western blot assays. (**H,I**) CCK8 assays exhibited the proliferative abilities of LC cells in different groups. LC, liver cancer; si-NCBP2, small interfering RNA targeting NCBP2; OE, overexpression; *P < 0.05, **P < 0.01, ***P < 0.001.

As shown in Table 2, there have been several studies that probed into the roles of m7G signature genes in multiple cancers. However, the biofunctions of NCBP2 in HCC remain elusive. In this context, NCBP2 was selected for further investigation. We first detected the NCBP2 expression in clinical specimens through RT-qPCR and western blot tests. RT-qPCR results showed that the mRNA expression of NCBP2 in HCC tissues was significantly higher than that in adjacent normal tissues (Figure 8A). The western blot detection on four pairs of clinical specimens also confirmed this expressive tendency. The protein expression levels of NCBP2 in tumor tissues were markedly upregulated compared to those in normal tissues (Figure 8B).

**Table 2:** Main roles of m7G signature genes in multiple cancers.

m7G signature gene	Study	Cancer type	Main function
WDR4	PMID: 34371184	LC, ICC	Promote cancer progression
	PMID: 34352206		
EIF4E	PMID: 33975880	BC, HCC	Support immune suppression, cancer metastasis, and drug resistance
	PMID: 33783376		
NCBP1	PMID: 31448526	LUAD	Promote cancer progression
NCBP2	PMID: 30250529	LC	Only bioinformatic prediction
NUDT1	PMID: 29075149	GC, LUAD	Promote cancer progression
	PMID: 21289483		

*BC, breast cancer; GC, gastric cancer; HCC, hepatocellular carcinoma; ICC, intrahepatic cholangiocarcinoma; LC, lung cancer; LUAD, lung adenocarcinoma; m7G, N7-methylguanosine .*

**Figure 9:**
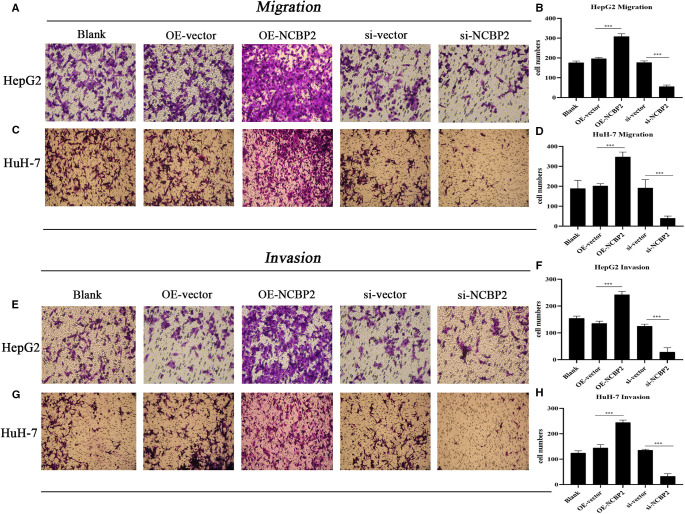
Overexpression of NCBP2 promotes the migration and invasion of LC cells. (**A,B**) Results of transwell migration assays in HepG2 and HuH-7 cells. (**C,D**) Migrative cell numbers in different experimental groups. (**E–G**) Results of transwell invasion assays in HepG2 and HuH-7 cells. (**F,H**) Invasive cell numbers in different experimental groups. *P < 0.05, **P < 0.01, ***P < 0.001.

NCBP2 was also significantly overexpressed in hepatoma cells (HepG2 and HuH-7) compared to that in normal liver epithelial cells (THLE-3) (Figure 8C). Meanwhile, we observed that si-NCBP2 and OE-NCBP2 could effectively manipulate the mRNA and protein expression levels of NCBP2 (Figures 8D–G). CCK8 assays revealed that overexpression of NCBP2 promoted the proliferation of hepatoma cells (Figures 8H,I). Inversely, silencing NCBP2 led to a decreased tendency (Figures 8H,I). Next, we applied transwell assays to evaluate the migrative and invasive abilities of hepatoma cells. Overexpression of NCBP2 was enhanced, whereas blocking NCBP2 expression suppressed the migration of hepatoma cells (Figures 9A–D). Similar alterations were observed in cellular invasive ability. Overexpression of NCBP2 increased the number of cells that penetrated Matrigel (Figures 9E–H). However, silencing NCBP2 retarded the invasive process (Figures 9E–H).

### Comparison Between Five Existing HCC Signatures and Our m7G Risk Signature

Given that there have been some studies constructing signatures for HCC clinical assessments, we analyzed the similarities and differences between five existing signatures and ours (24–28) (Table 3). It was not difficult to perceive that our novel m7G model exhibited several strengths. First, we applied three validation cohorts to test the prognostic value of the m7G risk signature, which effectively assured its applicable scope due to the maximum sample size. Second, our risk signature (AUC = 0.733) presented a moderate preponderance in the predictive accuracy compared to the signatures of Wu et al*.* (AUC = 0.698) (28), Gao et al*.* (AUC = 0.614) (25), and He et al. (AUC = 0.705) (26). Although our model was not as accurate as the models of Deng et al. (AUC = 0.757) (24) and Shen et al. (AUC = 0.737) (27), the gene number of our signature is much smaller than their ones (5 vs. 19 and 10, respectively). Considering this fact, the calculation of the m7G risk score based on our model was more accessible, which was more beneficial for clinical practice. Third, other studies did not investigate the biofunctions of core signature genes in HCC through an experimental approach, which attenuated the credibility and value of the models. Nevertheless, in the present study, we ascertained the pro-oncogenic capacities of NCBP2 in HCC for the first time, providing new insights into HCC treatment. Briefly, our m7G signature was reliable and had great potential in the clinical assessment of HCC.

**Table 3:** Comparison between five related research studies and our study.

Study	PMID	Model type	Number of signature genes	Validation cohort	Predictive accuracy (Mean value)	Experimental validation
Wu et al.	34869364	Pyroptosis	2	ICGC (*n* = 231)	0.698	NA
Deng et al.	33686959	Glycolysis	19	ICGC (*n* = 231)	0.757	NA
Gao et al.	33789609	Immune-related	7	Internal validation	0.614	NA
He et al.	34257161	FA metabolism	6	ICGC (*n* = 231)	0.705	NA
Shen et al.	34820373	Autophagy	10	TCGA (*n* = 342)	0.737	NA
Our	NA	m7G	5	GSE14520 (*n* = 221)	0.733	Biofunctions of NCBP2
				GSE116174 (*n* = 64)		
				ICGC (*n* = 231)		

*FA, fatty acid; m7G, N7-methylguanosine; NA, not available.*

## Discussion

HCC is a common abdominal tumor with a poor prognosis, leading to more than 30,000 deaths across the United States in 2021 (1). Owing to the easy metastasis and the limited efficacy of current therapeutic approaches, finding breakthrough treatment and establishing an accurate prognostic assessment system are extremely meaningful. m7G is a pivotal RNA modification that widely exists in various types of RNA, especially in tRNA (29). Due to its stimulative effects on the stability of RNA, it has been demonstrated that the m7G process could promote cancer progression by upregulating the expressions of oncogenic genes (16, 30). In the present study, we preliminarily probed the functions of m7G regulatory genes in HCC.

Accurate prognostic evaluation has pivotal instructive significance for making therapeutic strategies. Regrettably, the current prognostic model has some defects. Beumer's team has reported that the American Joint Committee on Cancer (AJCC) system failed to operate in an external cohort (31). Meanwhile, its predictive C-index was less than 0.7 (31). Hence, utilizing the TNM or AJCC prognostic system could not completely meet the demand of accurately predicting the survival outcomes. In this work, the novel m7G risk score not only increased the clinical making-decision benefit of TNM and clinical stage models but also elevated their predicted accuracy. All of these findings highlighted that the m7G risk score was a critical supplement for the prognostic assessment of HCC.

Due to participating in the maturation and response function of immune cells, RNA methylation profoundly affects the TIM (12). For instance, m7G recognition is responsible for driving the retinoic acid-inducible gene-I (RIG-I)-mediated innate immune (32). In the current study, we confirmed that the m7G risk score was tightly associated with the TIM of HCC. Especially, high m7G risk obviously decreased the immune abundance of CD8+ T cells, whereas it increased that of Tregs and macrophages. As is well known, CD8+ T cells have potent abilities to eradicate tumor cells through the Fas/FasL pathway (33). However, Tregs and macrophages could make immunologic barriers against CD8+ T cell-mediated antitumor immune response (34). Therefore, high m7G risk may herald that the antitumor immune process is arrested.

Cancer progression is commonly accompanied by metabolic reprogramming. Glycolysis, also named the “Warburg effect”, acts as a core hallmark of cancer metabolism. It is now established that glycolysis contributes to tumor growth, drug resistance, and immune escape (35–37). For example, the glycolysis process was found to be remarkably upregulated in RCC samples, and its core regulatory genes PLOD1, PLOD2, and CD44 could promote the proliferation of renal cancer cells (38). Through GESA analyses, we observed that high m7G risk resulted in a similar metabolic alteration; for example, the glycolysis process was significantly enriched in the HCC samples with high m7G risk. We speculated the m7G signature gene, EIF4E, may act as the intrinsic driving force. Evidence has emerged that the mTORC1/EIF4E/HIF-1α pathway is capable of mediating glycolysis (39). Moreover, EIF4E-mediated glycolysis has been proven to be a critical link in PAAD development (40).

There has been some research to unravel the roles of some m7G signature genes in cancer progression. For example, Zeng et al. revealed that WDR4, the core subunit of the m7G functional complex, extensively affected the cancer immunity in pan cancer (41). Silencing METTL1 or WDR4 inhibits the malignant behavior of HCC cells (42). NCBP1 promotes the development of LUAD through upregulating CUL4B (43). Herein, we focused on the lesser-studied NCBP2. NCBP2 encodes a product that is a component of the nuclear cap-binding protein complex (CBC), which binds to the monomethylated 5′ cap of nascent pre-mRNA in the nucleoplasm (44). In the present study, we ascertained its tumorigenicity in HCC cells for the first time. Blocking NCBP2 markedly inhibited the proliferation, migration, and invasion of HCC cells, which indicated that NCBP2 is a potential therapeutic target for HCC.

Notably, there are several limitations to this study. First, the m7G risk signature has not been validated in a real clinical cohort. Second, we did not compare the differences in the m7G modification intensity between high- and low-m7G-risk groups. Third, the oncogenic capacities of NCBP2 have not been verified in a xenograft tumor model of nude mice. Fourth, the immune effects of NCBP2 in HCC were not confirmed by experiments in vitro. These issues warrant further research.

## Conclusions

HCC is a common digestive tumor bringing a great health burden on patients. As one of the most common RNA modifications, m7G shows promising potential to improve cancer treatment. In view of this, we constructed a novel m7G risk signature through lasso regression analysis. The m7G risk score exhibited pivotal functions in the prognostic assessment of HCC. Meanwhile, it extremely improved the predictive performance of the TNM staging or AJCC system. Moreover, high m7G risk was detrimental to antitumor immune but increased the enrichments of glycolysis metabolism. Of note, we ascertained the cancer-promoting abilities of NCBP2 in HCC for the first time. In conclusion, these findings provide new clues for prognostic assessment and treatment of HCC.

## Data Availability

The original contributions presented in the study are included in the article/Supplementary Material, further inquiries can be directed to the corresponding author/s
