# Supplementary figures and images for: N7-Methylguanosine Regulatory Genes Profoundly Affect the Prognosis, Progression, and Antitumor Immune Response of Hepatocellular Carcinoma
Source: Front Surg. 2022 Jun 16;9:893977. doi: 10.3389/fsurg.2022.893977 (PMC9246272; doi:10.3389/fsurg.2022.893977)

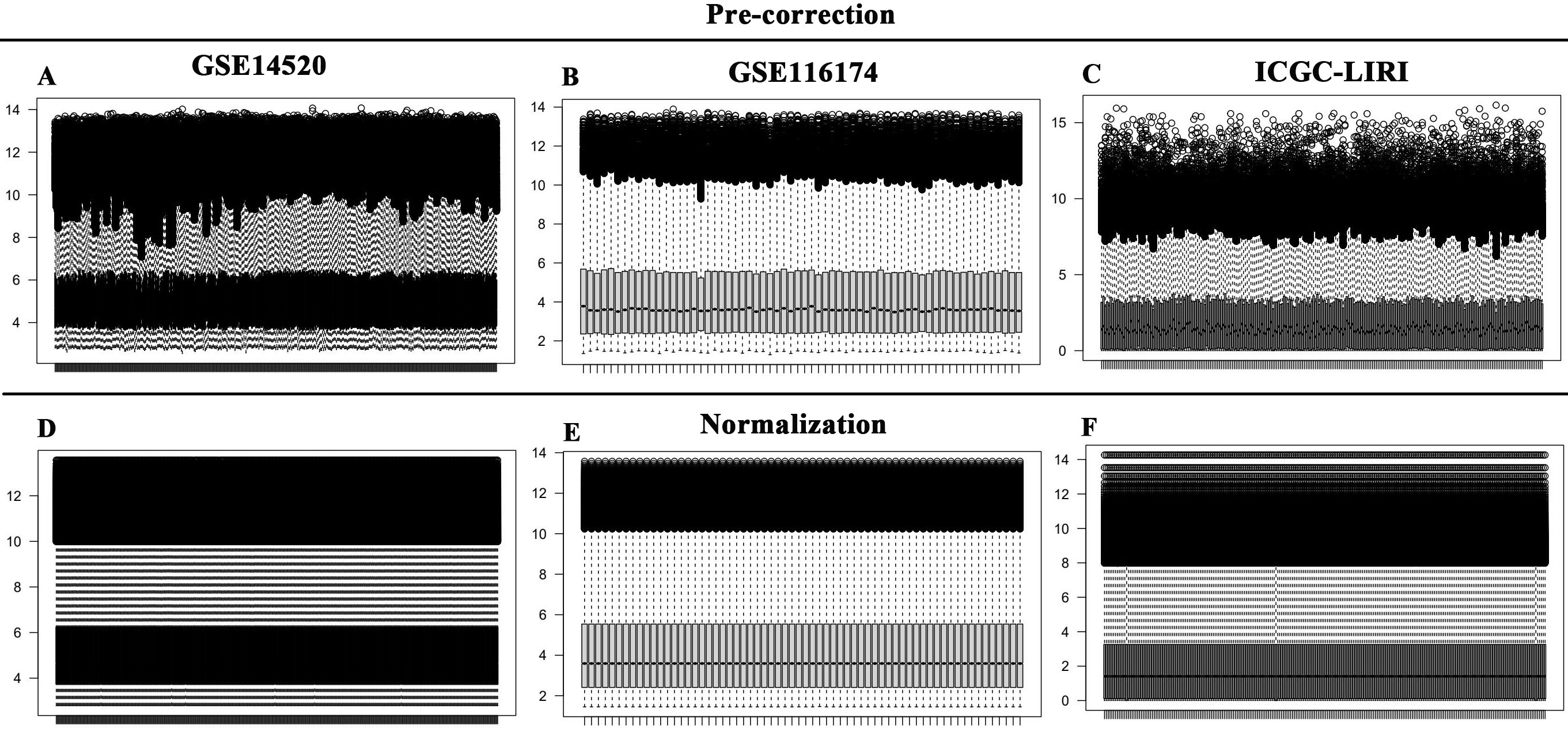

Supplement: Supplementary file 1 [file Supplementary_figure_1.jpg]

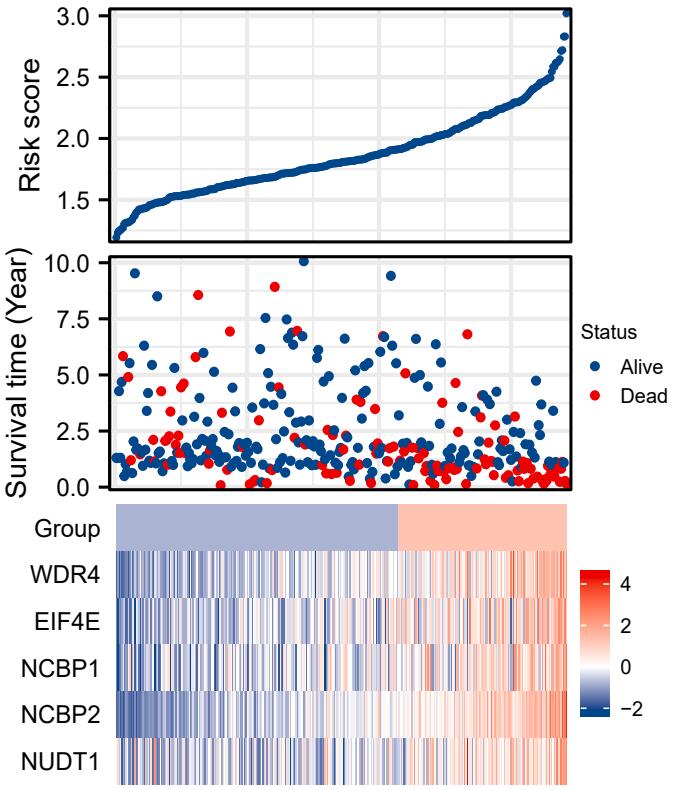

Supplement: Supplementary file 2 [file Supplementary_figure_2.jpg]

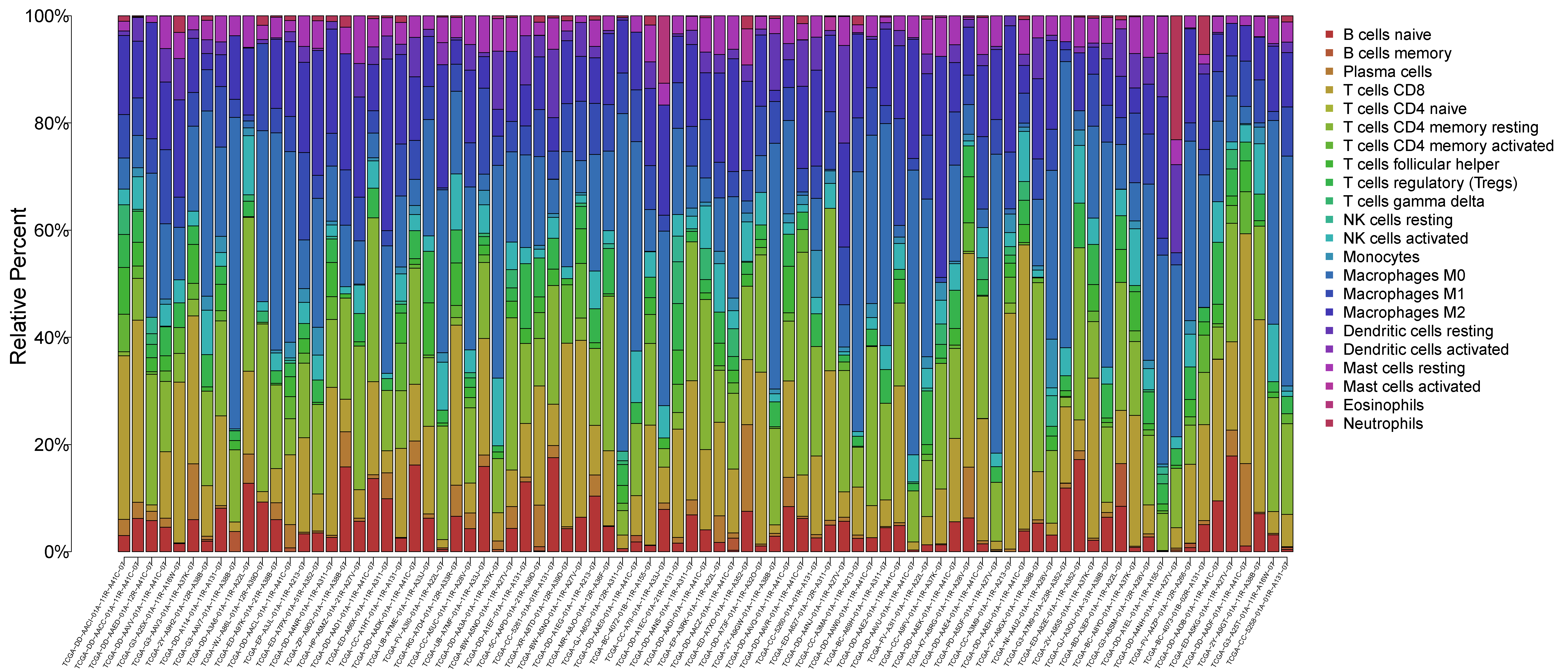

Supplement: Supplementary file 3 [file Supplementary_figure_3.jpg]
