# Supplementary material for: N7-Methylguanosine Regulatory Genes Profoundly Affect the Prognosis, Progression, and Antitumor Immune Response of Hepatocellular Carcinoma
Source: Front Surg. 2022 Jun 16;9:893977. doi: 10.3389/fsurg.2022.893977 (PMC9246272; doi:10.3389/fsurg.2022.893977)
Supplement: Supplementary file 5 [file Supplementary_table_2.docx]

Supplementary Table 2. The clinical characteristics of two GEO cohorts

| Items | GSE116174 | GSE14520 |
| --- | --- | --- |
| Sample size | 64 | 221 |
| Survival status |  |  |
| Dead | 27 | 85 |
| Alive | 37 | 136 |
| Age |  |  |
| ＜60 | 44 | 178 |
| ≥60 | 20 | 43 |
| Clinical stage |  |  |
| Stage I | 8 | 93 |
| Stage II | 45 | 77 |
| Stage III | 11 | 49 |
| Stage IV | 0 | 0 |
| Unknown | 0 | 2 |
| TNM-staging and Histological grade | NA | NA |

NA, not available.
