# Supplementary material for: N7-Methylguanosine Regulatory Genes Profoundly Affect the Prognosis, Progression, and Antitumor Immune Response of Hepatocellular Carcinoma
Source: Front Surg. 2022 Jun 16;9:893977. doi: 10.3389/fsurg.2022.893977 (PMC9246272; doi:10.3389/fsurg.2022.893977)
Supplement: Supplementary file 6 [file Supplementary_table_3.docx]

Supplementary table 3. Three core m7G gene set from MSigDB database

| Names | Gene counts | Description |
| --- | --- | --- |
| GOMF m7G 5-PPPN Diphosphatase Activity | 12 | Catalysis of the reaction: 7-methylguanosine 5'-triphospho-5'-polynucleotide + H2O = 7-methylguanosine 5'-phosphate + polynucleotide |
| GOMF RNA 7-Methylguanosine Cap Binding | 13 | Binding to a 7-methylguanosine group added cotranscriptionally to the 5' end of RNA molecules transcribed by polymerase II |
| GOMF RNA Cap Binding | 20 | Binding to a 7-methylguanosine (m7G) group or derivative located at the 5' end of an RNA molecule. |

m7G, 7-methylguanosine.
