# Supplementary material for: N7-Methylguanosine Regulatory Genes Profoundly Affect the Prognosis, Progression, and Antitumor Immune Response of Hepatocellular Carcinoma
Source: Front Surg. 2022 Jun 16;9:893977. doi: 10.3389/fsurg.2022.893977 (PMC9246272; doi:10.3389/fsurg.2022.893977)
Supplement: Supplementary file 7 [file Supplementary_table_4.docx]

Supplementary table 4. The pivotal m7G regulatory genes

| METTL1 | EIF3D | GEMIN5 | NCBP3 | NUDT16L1 |
| --- | --- | --- | --- | --- |
| WDR4 | EIF4A1 | IFIT5 | SNUPN | NUDT3 |
| NSUN2 | EIF4E | LARP1 | DCP2 | NUDT4 |
| AGO2 | EIF4E1B | LSM1 | NUDT1 | NUDT4B |
| CYFIP1 | EIF4E2 | NCBP1 | NUDT10 | NUDT5 |
| CYFIP2 | EIF4E3 | NCBP2 | NUDT11 | NUDT7 |
| DCPS | EIF4G3 | NCBP2L | NUDT16 |  |
