# Supplementary material for: N7-Methylguanosine Regulatory Genes Profoundly Affect the Prognosis, Progression, and Antitumor Immune Response of Hepatocellular Carcinoma
Source: Front Surg. 2022 Jun 16;9:893977. doi: 10.3389/fsurg.2022.893977 (PMC9246272; doi:10.3389/fsurg.2022.893977)
Supplement: Supplementary file 9 [file Supplementary_table_5.docx]

Supplementary table 5. The detailed information of gene sets used for GSEA

| Names | Gene counts | Description |
| --- | --- | --- |
| Hallmark Glycolysis | 200 | Genes encoding proteins involved in glycolysis and gluconeogenesis. |
| GO Glycolytic Process | 106 | Glycolysis begins with the metabolism of a carbohydrate to generate products that can enter the pathway and ends with the production of pyruvate. Pyruvate may be converted to acetyl-coenzyme A, ethanol, lactate, or other small molecules. |
| WP Ferroptosis | 65 | Ferroptosis |
| Reactome Pyroptosis | 27 | Pyroptosis |
