# Supplementary material for: N7-Methylguanosine Regulatory Genes Profoundly Affect the Prognosis, Progression, and Antitumor Immune Response of Hepatocellular Carcinoma
Source: Front Surg. 2022 Jun 16;9:893977. doi: 10.3389/fsurg.2022.893977 (PMC9246272; doi:10.3389/fsurg.2022.893977)
Supplement: Supplementary file 10 [file Supplementary_table_6.docx]

Supplementary Table 6. The primer lists

| Gene | Primer | Sequence (5' -> 3') |
| --- | --- | --- |
| NCBP2 | Forward | 5′- CTCTGCACTATGTCGGGTGG-3′ |
|  | Reverse | 5′- TGGCGTTTTCCGCATAGCTT-3 |
| GAPDH | Forward | 5'‐GTCGCCAGCCGAGCCACATC‐3 |
|  | Reverse | 5'‐CCAGGCGCCCAATACGACCA‐3' |
